# Abnormalities in the Polysomnographic, Adenosine and Metabolic Response to Sleep Deprivation in an Animal Model of Hyperammonemia

**DOI:** 10.3389/fphys.2017.00636

**Published:** 2017-08-31

**Authors:** Selena Marini, Olena Santangeli, Pirjo Saarelainen, Benita Middleton, Namrata Chowdhury, Debra J. Skene, Rodolfo Costa, Tarja Porkka-Heiskanen, Sara Montagnese

**Affiliations:** ^1^Department of Biology, University of Padua Padua, Italy; ^2^Department of Physiology, Institute of Biomedicine and Physiology, University of Helsinki Helsinki, Finland; ^3^Chronobiology, Faculty of Health and Medical Sciences, University of Surrey Guildford, United Kingdom; ^4^Department of Medicine, University of Padua Padua, Italy

**Keywords:** hyperammonemia, hepatic encephalopathy, sleep homeostasis, adenosine, metabolomics/metabolic profiling

## Abstract

Patients with liver cirrhosis can develop hyperammonemia and hepatic encephalopathy (HE), accompanied by pronounced daytime sleepiness. Previous studies with healthy volunteers show that experimental increase in blood ammonium levels increases sleepiness and slows the waking electroencephalogram. As ammonium increases adenosine levels *in vitro*, and adenosine is a known regulator of sleep/wake homeostasis, we hypothesized that the sleepiness-inducing effect of ammonium is mediated by adenosine. Eight adult male Wistar rats were fed with an ammonium-enriched diet for 4 weeks; eight rats on standard diet served as controls. Each animal was implanted with electroencephalography/electromyography (EEG/EMG) electrodes and a microdialysis probe. Sleep EEG recording and cerebral microdialysis were carried out at baseline and after 6 h of sleep deprivation. Adenosine and metabolite levels were measured by high-performance liquid chromatography (HPLC) and targeted LC/MS metabolomics, respectively. Baseline adenosine and metabolite levels (12 of 16 amino acids, taurine, t4-hydroxy-proline, and acetylcarnitine) were lower in hyperammonemic animals, while putrescine was higher. After sleep deprivation, hyperammonemic animals exhibited a larger increase in adenosine levels, and a number of metabolites showed a different time-course in the two groups. In both groups the recovery period was characterized by a significant decrease in wakefulness/increase in NREM and REM sleep. However, while control animals exhibited a gradual compensatory effect, hyperammonemic animals showed a significantly shorter recovery phase. In conclusion, the adenosine/metabolite/EEG response to sleep deprivation was modulated by hyperammonemia, suggesting that ammonia affects homeostatic sleep regulation and its metabolic correlates.

## Introduction

Hepatic encephalopathy (HE) is a syndrome of neurological and psychiatric symptoms which complicates liver cirrhosis. HE is due to gut-derived neurotoxins, such as ammonia, which escape hepatic detoxification and reach the brain through the systemic circulation (Sherlock et al., 1954; Vilstrup et al., 2014). HE is often observed after upper gastro-intestinal bleeding, because the blood is ingested and the amino acids absorbed by the small intestine are rapidly oxidized, with a sharp increase in ammonium levels. In humans, this situation can be simulated by oral administration of a mixture of amino acids of comparable composition to that of hemoglobin (amino acid challenge; Douglass et al., 2001).

HE patients show excessive daytime somnolence, which has been connected to elevated ammonium levels (Bersagliere et al., 2012). We have previously shown that an amino acid challenge results in increased subjective sleepiness both in healthy volunteers and in patients with cirrhosis (Bersagliere et al., 2012). A more detailed EEG analysis showed increased power and increase in large amplitude waves during waking, compatible with the self-reported feeling of sleepiness (Bersagliere et al., 2012). However, while after an amino acid challenge healthy volunteers slept longer and deeper, patients with cirrhosis, who are chronically hyperammonemic, seemed unable to convert sleepiness into restorative, deep sleep (Bersagliere et al., 2012). The mechanisms by which hyperammonemia induces sleepiness, and in experimental participants increases sleep, have remained unexplored. Also the question regarding the difference in the sleep response between the patients and healthy volunteers has remained open.

Adenosine is one of the known sleep-inducing molecules, which is particularly involved in the mediation of sleep homeostasis (Brown et al., 2012; Porkka-Heiskanen, 2013). Cerebral levels of extracellular adenosine increase during wakefulness and decrease during sleep (Porkka-Heiskanen et al., 1997, 2000), and extracellular levels of adenosine increase in the basal forebrain during sleep deprivation (Porkka-Heiskanen et al., 1997). As an acute dose of caffeine, an adenosine receptor antagonist (Ribeiro and Sebastião, 2010), counteracts the effect of induced hyperammonemia in healthy volunteers (Casula et al., 2015), we hypothesized that the sleepiness/sleep-inducing effects of hyperammonemia are mediated, at least partly, via adenosine.

To test this hypothesis, we measured adenosine levels from brain microdialysates collected from rats that were made hyperammonemic and controls during waking, sleep, sleep deprivation, and recovery sleep.

To further explore the effect of hyperammonemia, we also measured metabolites in microdialysate from hyperammonemic and control animals before and after sleep deprivation using a targeted metabolomics approach (Skene et al., 2017).

## Methods

Adult male Wistar rats (250–280 g) were fed an ammonium-enriched diet (Felipo et al., 1988) *ad libitum* for 4 weeks. A standard diet (Teklad Global 18% Protein Rodent Diet, Harlan) was mixed with ammonium acetate (20% w/w) and the appropriate amount of water to produce a homogenous mixture, which was subsequently air-dried (40°C for 2–3 days). Control rats were fed a standard diet prepared following the same procedure except for the addition of ammonium acetate.

Animals were kept under constant temperature (23–25°C) on a 12 h light–12 h dark cycle, with lights on from 09:00 to 21:00 h.

All animal procedures were approved by the University of Helsinki Ethical Committee for Animal Experiments and by the Regional Committee of the State Provincial Office and performed according to applicable national and European Union legislation.

### Ammonia levels

Fasting (3–4 h) capillary ammonia concentrations were measured during the fourth week using an Ammonia Checker (Menarini Diagnostics, Firenze, Italy). The pre-warmed foot was punctured with a 25G needle, 20 μl of blood obtained via a capillary tube and transferred to an *ad hoc* reagent strip. Ammonia concentrations were then determined by a reflectance meter (Huizenga et al., 1995).

### Surgery

Animals were habituated to handling at least 4 days prior to surgery, which was performed during the fourth week. Under general anesthesia, they were implanted with electroencephalography/electromyography (EEG/EMG) electrodes and a microdialysis guide cannula. They were anesthetized with isoflurane (IsoFlo®Vet 100%, Abbott Laboratories Ltd, England) (5% induction, 2% maintenance) and placed in a stereotaxic device. Before the surgery they were injected with buprenorphine (Temgesic®, Indivior UK Limited, Slough, UK, 0.05 mg/kg, s.c.). After exposing, cleaning and disinfecting the skull bone, two gold-coated screws were fitted into the skull for frontal-parietal epidural bipolar recording of the EEG, while two silver wire electrodes were inserted into the neck muscles for EMG recording. A unilateral guide cannula (CMA11 Guide Cannula; CMA/Microdialysis, Stockholm, Sweden) was implanted targeting the basal forebrain cholinergic area (Porkka-Heiskanen et al., 2000) for subsequent insertion of the microdialysis probe. Electrodes and guide cannula were fixed to the skull with acrylic dental cement. After surgery, animals were individually housed in open Plexiglas boxes, connected to the recording cables through swivels allowing them to move freely. Rats were left for adaptation and recovery for at least 5 days.

### *In vivo* microdialysis and EEG/EMG recording

**Figure 1:**
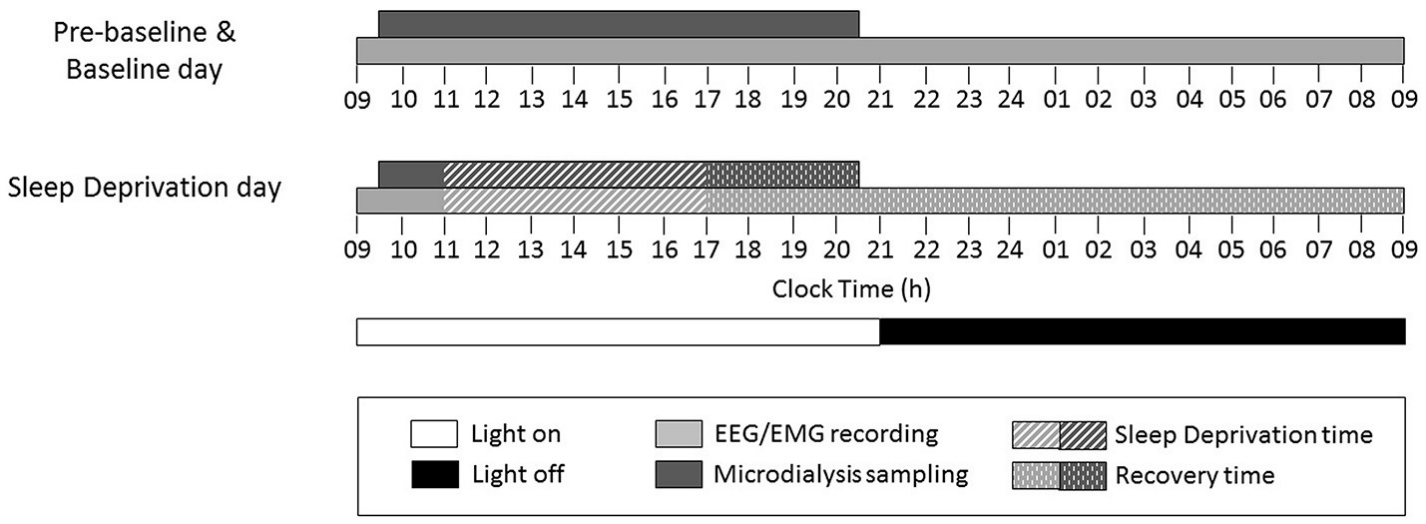
Study design and timing of experimental procedures on each study day.

Each rat underwent adenosine sample collection by *in vivo* microdialysis, and EEG/EMG recording during three consecutive days [pre-baseline (pre-BL), baseline (BL), and sleep deprivation (SD) day] (Figure 1). A microdialysis probe (CMA11 Microdialysis probe; 14 mm shaft length, 2 mm dialyzing membrane length, 0.24 mm diameter; CMA/Microdialysis) was inserted through the guide cannula into the basal forebrain (Porkka-Heiskanen et al., 2000) and inlet and outlet tubing (1.5 m pieces of fluorinated ethylene propylene tubing; CMA/Microdialysis) connected to the probe the day before the beginning of the experiment. Artificial cerebrospinal fluid (NaCl 147 mM, KCl 3 mM, CaCl_2_ 1.2 mM, MgCl_2_ 1 mM, pH 7.2) was continuously perfused at a flow rate of 1 μl/min. Thirty minute dialysate samples were automatically collected for 11 h during the light period from 09:30 to 20:30 h and stored at −20°C.1.

EEG/EMG recordings were sampled at 277.78 Hz with a resolution of 8 mV/bit. Signals were amplified (gain EEG: 5,000; EMG: 2,000), analogically filtered (EEG: 0.3 Hz high-pass, 100 Hz low-pass; EMG: 10 Hz high-pass, 100 Hz low-pass), monitored on-line during the experiments and stored on a computer equipped with the Spike 2 software (version 5.19; CED, Cambridge, UK) for off-line analysis.

### Sleep deprivation

On the third day, animals were sleep-deprived for 6 h, starting at 11:00 h (Figure 1), by gentle handling (Franken et al., 1991), which includes presentation of new objects into the cage and tactile stimulation, such as light touches of a brush/hand. After sleep deprivation, microdialysis sampling and EEG/EMG recording were continued and animals left undisturbed to allow for recovery sleep (Figure 1).

### Probe tips location

**Figure 2:**
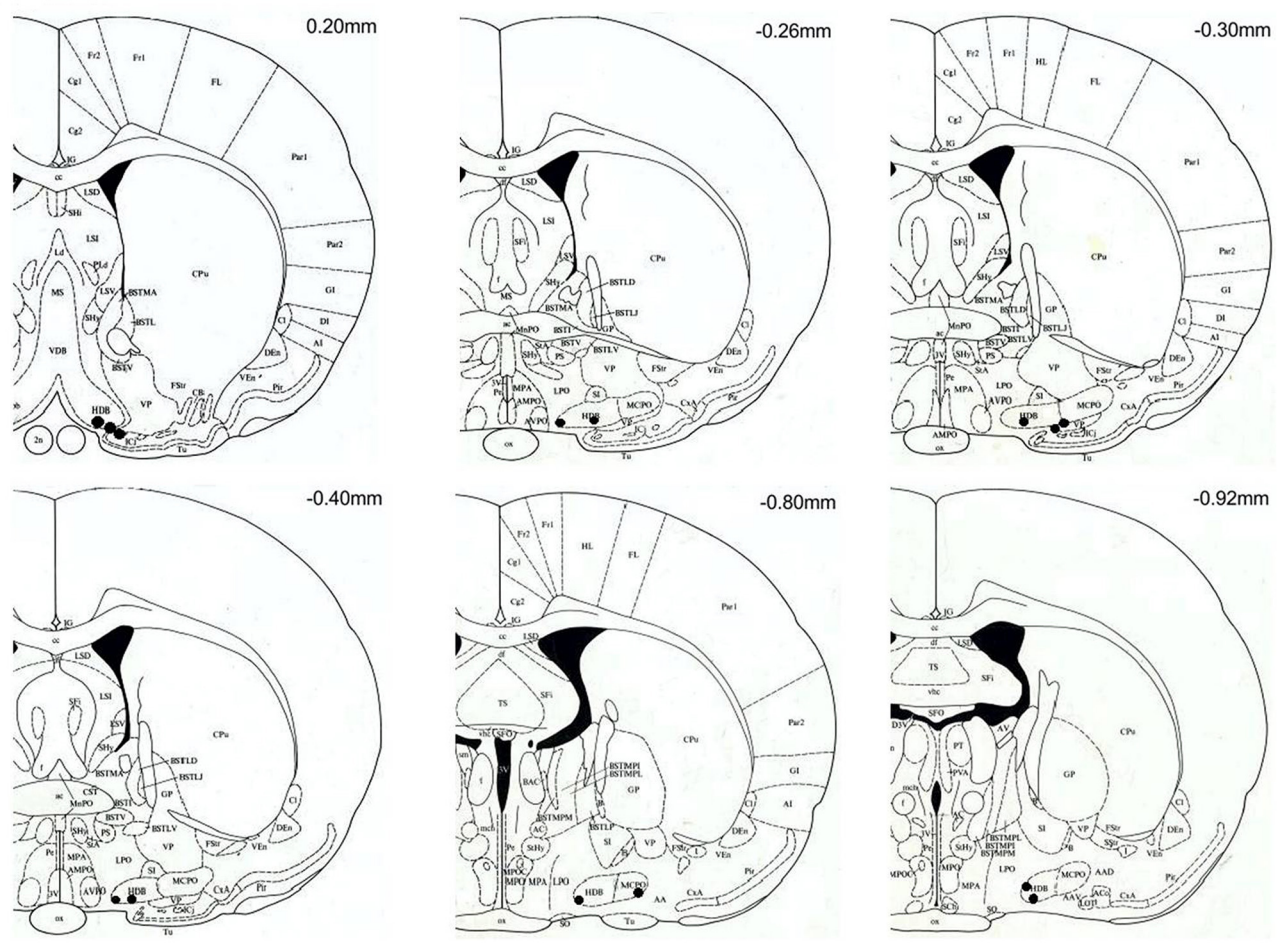
Coronal brain sections (modified from Paxinos and Watson, 1998) with black spots marking the location of the microdialysis probe tips. All probe tips were located between anterior-posterior levels of +20 and −92 mm with respect to bregma. Target areas include: horizontal diagonal band of Broca (HDB), substantia innominata (SI), magnocellular preoptic area (MCPO), lateral preoptic area (LPO) and the basal nucleus of Meynert (B). The location of probes with tips outside the target area are not shown, and the corresponding rats (*n* = 2, both on the ammonia-enriched diet) were excluded from the analysis.

At the end of the recovery period, rats were sacrificed with a lethal dose of pentobarbital (Mebunat 60 mg/kg). The location of the tips of the probes was marked by injecting color ink through a modified microdialysis probe. Brains were removed and immediately frozen. Twenty micrometer coronal sections were cut using a cryostat, mounted on a glass plate and stained with toluidine blue. The location of each probe was verified by visual inspection under a light microscope, using a rat brain atlas (Paxinos and Watson, 1998). Only those rats with probe locations in the close vicinity of the target area, including the horizontal diagonal band of Broca, substantia innominata, magnocellular preoptic area, lateral preoptic area and the basal nucleus of Meynert, were included in subsequent analyses (Figure 2).

### Adenosine measurement

The dialysate samples were analyzed with a microbore high-performance liquid chromatography (HPLC) system coupled to a fluorescent detector (Waters 2,475), as previously described (Kalinchuk et al., 2015). The mobile phase, consisting of ammonium acetate 50 mM, tetrabutyl-ammonium hydrogen sulfate 0.2 mM, EDTA 1 mM in 15% of MeOH, pH 5.6, was passed through the column at a flow rate of 1 ml/min. Ten microliters of the first 30 min sample of every hour from BL and SD days were injected through a Kinetex® C18 column (100A, 150 × 4.60 mm, particle size: 5 micron, Phenomenex, USA), coupled with a pre-column (KJO-4282, Phenomenex, USA). Adenosine was detected at a wavelength of 460 nm and its concentrations were determined by comparing sample peak areas with those of standards (AMP, adenosine, cAMP, cdAMP, Sigma-Aldrich) using LabSolutions software (Shimadzu). Detection limit: 0.1 nM; linear range of the assay: 0.1–50 nM; coefficient of variation < 10%. Any outliers were determined and treated according to a modified Thompson tau Method.

One of the main features of this microdialysis technique is that the sample is collected through a membrane, which cannot be penetrated by large molecules, i.e., enzymes (Porkka-Heiskanen et al., 1997). Thus, the degradation of adenosine in the collected microdialysis samples is completely dependent on the stability of the molecular structure, not on the degrading enzymes (as opposed to what happens in tissue samples, where adenosine is instantly metabolized). The recovery rate of adenosine, measured from known concentrations of adenosine solutions, is of the order of 10%, depending on flow rate, membrane, temperature and the concentration of the original solution.

### Targeted LC/MS metabolomics

A subset of the dialysate samples (*n* = 69) were analyzed in 5 control and 5 hyperammonaemic animals using the Absolute***IDQ***® p180 targeted metabolomics kit (Biocrates Life Sciences AG, Innsbruck, Austria) on a Waters Xevo TQ-S mass spectrometer (MS) coupled to an Acquity UPLC system (Waters Corporation, Milford, MA, USA; Davies et al., 2014). We took two samples per each animal during BL day: one at ZT3.5 and another one at ZT8, which correspond to the time points of beginning and middle of SD during SD days. This was done to evaluate metabolites during SD (i.e., BL vs. SD analysis). Then we took three samples per each animal during SD: at ZT4, ZT5.5 and ZT7.5. These correspond to the beginning (2 h after beginning of SD when, according to our experience, changes start to occur, i.e., adenosine rise), middle and the end of the SD. Finally, two samples of recovery period were taken from each animal: at ZT8.5 and ZT9.5 to evaluate metabolites immediately after finishing SD (during the first hour of recovery), and later on, closer to the end of light period. The dialysate samples (10 μl) were prepared according to the manufacturer's instructions adding several stable isotope–labeled standards to the samples prior to derivatisation and extraction. Sample order was randomized and all samples were run on a single 96-well plate including 3 levels of quality controls. Up to 183 metabolites from 5 different compound classes (acylcarnitines, amino acids, biogenic amines, glycerophospholipids and sphingolipids) can be quantified using either LC/MS or flow injection analysis/MS. Metabolites were selected if the % valid or % semiquantitative values were above 60% (average 76%). In addition, putrescine (35%), acetylcarnitine (C2) (40%), DOPA (46%), and SDMA (49%) were included since their values were above zero but below the lower limit of quantification.

### Sleep-wake scoring

All EEG traces were digitally high-pass filtered (1.4 Hz cut-off). EEG recordings were then semi-automatically scored in 4 s epochs using AUTOSCORE script, version 1.7 (Rytkönen et al., 2011) and manually checked using the SLEEPSCORE script, version 1.01 (CED). Vigilance states were scored according to standard criteria (Kalinchuk et al., 2015). Briefly, NREM sleep was recognized by high-amplitude EEG associated with slow waves in the delta range (0.5–4 Hz) and low-voltage EMG; REM sleep was recognized by low-amplitude, high-frequency EEG associated with the absence of EMG except for whiskers and ears twitches, as well as EEG theta activity (4–9 Hz); wakefulness was recognized as low-amplitude, high-frequency EEG activity with high-voltage EMG. The time spent in wake/NREM/REM was calculated over the total recording time of BL day, and hourly values compared between the two groups. The number of state-specific bouts per hour and the bout length distribution over the 24-h BL day were also obtained. State-specific time during the 16 h of recovery of the SD day were compared to the corresponding values at baseline, on an hourly basis.

### Statistical analysis

Data are presented as means ± SD or SEM. Baseline ammonia and point adenosine/metabolite values were compared by the Student t or Mann-Whitney *U*-test, as appropriate. Baseline adenosine/metabolite values and differences between post-sleep deprivation and baseline values were analyzed by repeated measures ANOVA (factors: *time* and *group*); *p*-values were corrected for multiple comparisons. Differences (sleep deprivation-baseline) were chosen over ratios (sleep deprivation/baseline) because of the presence of zero values for wake/NREM/REM in several time bins. Empty cells, if any, were treated by standard missing values replacement and analyses were performed both prior to (missing out the pertinent time points) and after replacement. Multivariate analysis of the metabolite data was performed by principal component analysis (PCA) and orthogonal partial least squares discriminant analysis (OPLS-DA) validated by permutation testing (p ≤ 0.05), using SIMCA-P v12.0 software (Umetrics, Sweden). Differences in individual metabolite levels were analyzed in R version 3.1.2 using the linear models and ANOVA methods in the stats package. Linear models were fitted to the group (control or ammonia treated) and time (*n* = 7), with the animal as covariate. Significant differences for group, time and their interaction were determined using 2-way ANOVA. *P*-values were corrected for multiple comparisons according to the Benjamini-Hochberg False Discovery Rate (FDR). Metabolites were considered significant at a FDR cut off <0.05. For testing statistical significance, missing values were not taken into account.

## Results

Capillary ammonia levels were significantly higher in the modified diet compared to the control group (73 ± 25 vs. 42 ± 15 μg/dl, *p* = 0.02), confirming that the diet had produced the expected results. Rats fed the ammonium-enriched diet tended to gain less weight compared to control animals over the treatment period and while the group factor was not significant, an interaction was detected between group and time (*time: F* = 74.9, *p* < 0.001; *group: F* = 1.3, *p* = 0.271; time^*^group: F = 8.5, *p* < 0.001). *Post-hoc* comparisons were not significant at any time point; actual weights from week 2, when the two groups started to diverge, were as follows (mean ± SD; standard vs. ammonia-enriched diet): 392 ± 34 vs. 369 ± 34 at week 2; 411 ± 28 vs. 381 ± 28 at week 3; 420 ± 23 vs. 387 ± 20 at week 4.

### Spontaneous sleep-wake cycle

**Figure 3:**
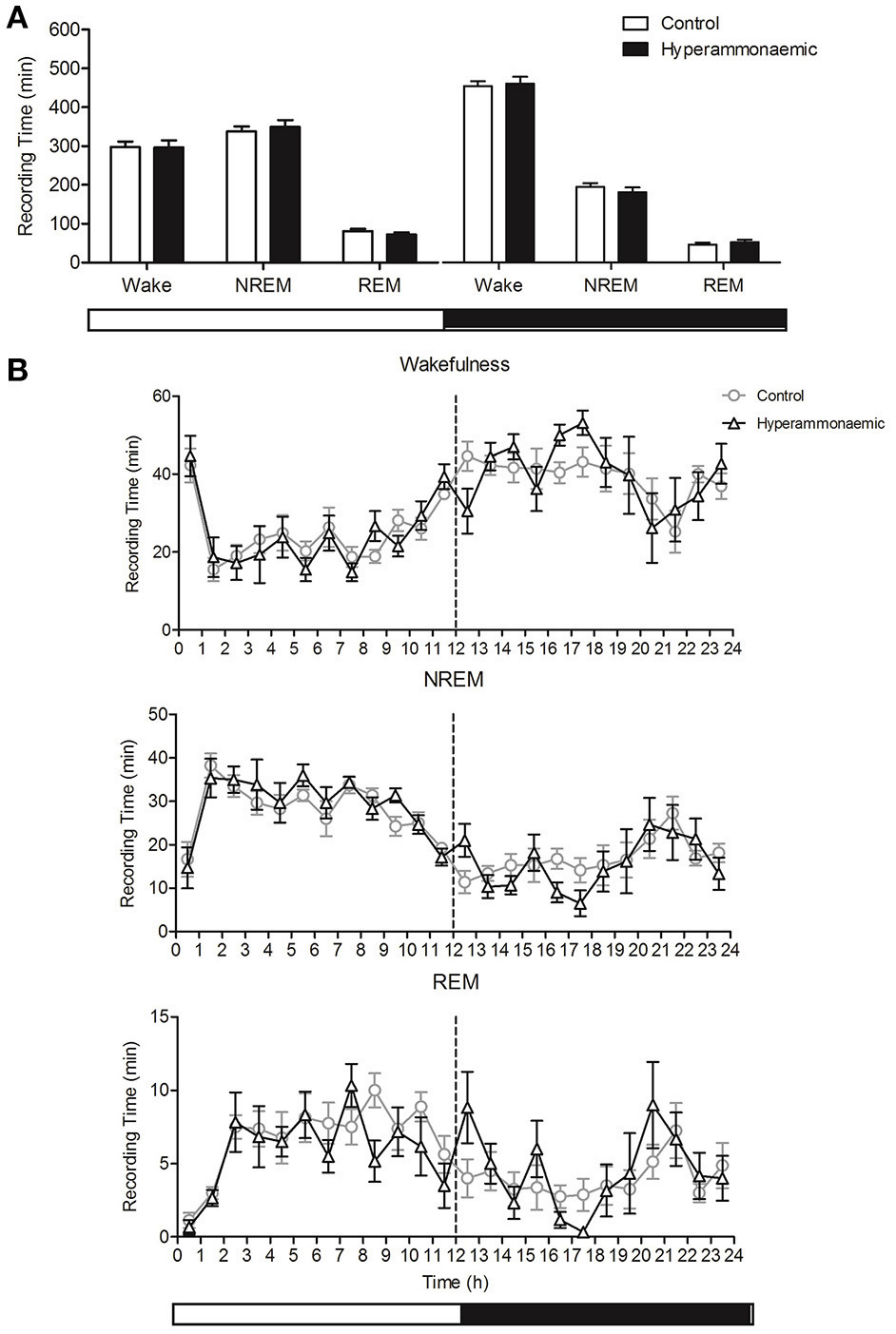
**(A)** Comparison of the total time (min) spent in Wake-NREM-REM state, in the light (left) and dark (right) period, between control (white bars) and hyperammonemic (black bars) animals, during the spontaneous sleep-wake cycle. **(B)** Comparison of Wakefulness, NREM and REM sleep profile (min/hour) between control (circles) and hyperammonemic (triangles) animals, during the spontaneous sleep-wake cycle.

Control and hyperammonemic rats spent comparable periods of time in wakefulness, NREM and REM sleep (Figure 3A). Similar state-specific time distributions over the 24 h were observed, with slight variations, particularly in REM sleep (Figure 3B). However, these did not impinge on total REM sleep time. The number of state-specific bouts per h and their length distribution were also comparable in control and hyperammonemic rats.

### Sleep deprivation-induced recovery sleep

**Figure 4:**
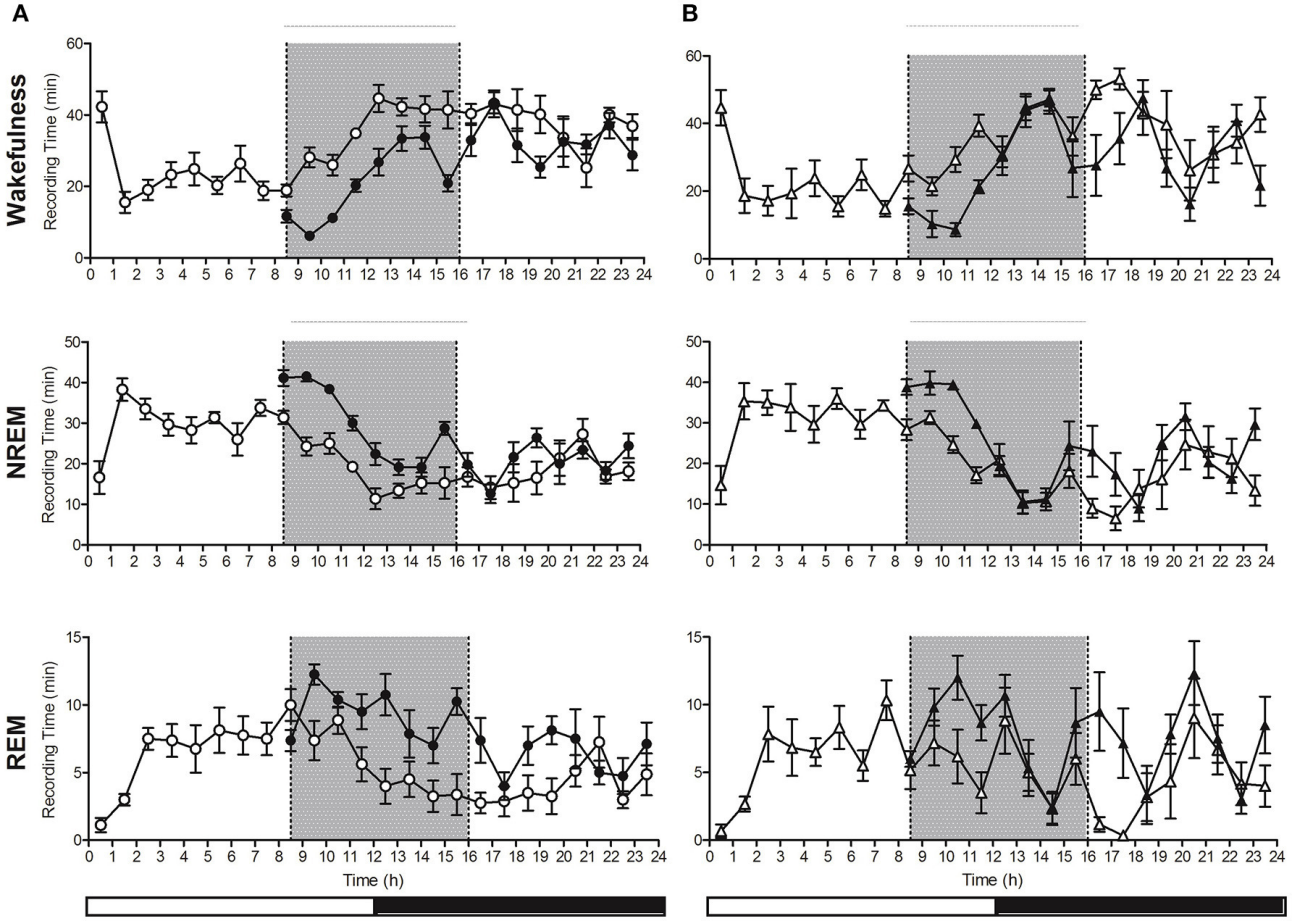
Hourly distribution of the time (minutes; mean ± SEM) spent in Wakefulness/NREM/REM at baseline (open symbols) and during the recovery period (closed symbols) in control (**A**; *n* = 8, circles) and hyperammonemic rats (**B**; *n* = 6, triangles). Analysis of recovery was performed on both the 9–16 h (gray portion of the graphs) and 9–24 h intervals. Statistical analysis of wakefulness, NREM and REM (presented in Table 1), was performed by comparing the differences between baseline and sleep deprivation in the control group **(A)** as well as baseline and sleep deprivation in the ammonium-treated group **(B)** [repeated measures ANOVA of the mathematical difference between baseline and recovery (in minutes), by group; Table 1].

**Figure 5:**
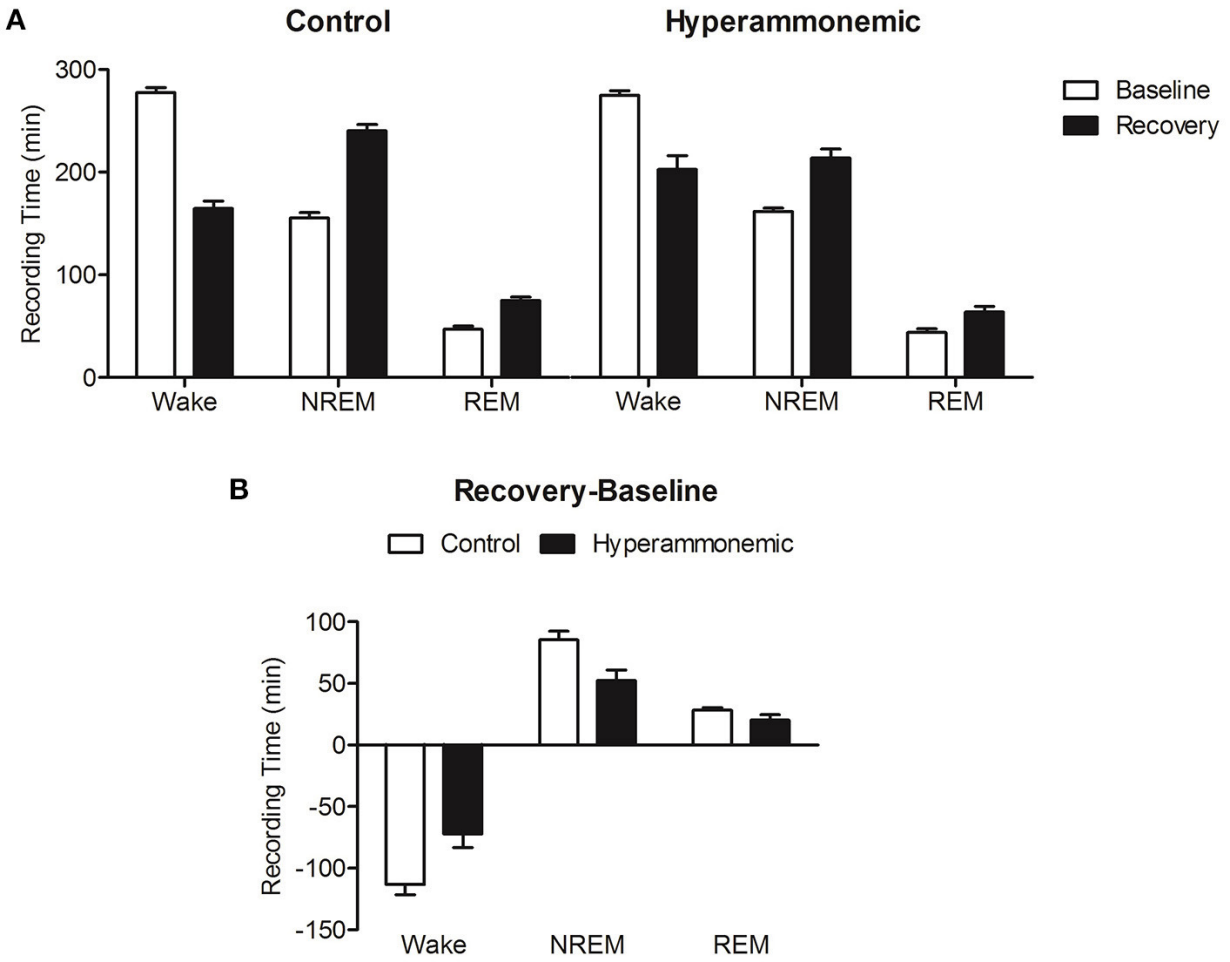
**(A)** Decrease in Wakefulness time (min) and increase in NREM and REM sleep time after sleep deprivation (9–16 h, black bars) with respect to baseline (white bars), in control (left) and hyperammonemic (right) animals. **(B)** Comparison of the differences (recovery minus baseline, 9–16 h) in Wakefulness, NREM and REM sleep time (min) between control (white bars) and hyperammonemic (black bars) animals.

Following sleep deprivation, a significant decrease in wakefulness and increase in NREM and REM sleep compared to baseline values was observed in both groups [Figure 4 (*n* = 8 control and *n* = 6 hyperammonaemic animals) and Figure 5]. Direct comparisons of the sleep time increase/wakefulness decrease showed similar average values in the control and hyperammonemia groups [wakefulness: −151 ± 9 min (−26%) vs. −142 ± 14 min (−24%); NREM: 105 ± 6 min (+35%) vs. 97 ± 9 min (+33%); REM: 46 ± 4 min (+58%) vs. 46 ± 6 min (+60%)].

**Table 1:** Statistical analysis of differences between baseline and recovery sleep in wakefulness, NREM and REM sleep in controls and in ammonium-treated animals over two time intervals: 9–16 and 9–24 h.

		**Recovery 9–16 h**			**Recovery 9–24 h**		
		***F***	***p***	***Post-hoc* (significant time points)**	***F***	***p***	***Post-hoc* (significant time points)**
Wakefulness	Time	2.9	**0.008**	/	2.3	**0.005**	/
	Group	9.4	**0.010**		0.3	0.572	
	Time^*^group	1.8	0.091		1.5	0.093	
NREM	Time	3.9	**0.001**	13, 14, 15	2.7	**0.001**	/
	Group	8.8	**0.012**		0.7	0.414	
	Time^*^group	1.3	0.252		1.4	0.134	
REM	Time	2.6	**0.019**	11, 13, 16	2.1	**0.011**	17
	Group	3.0	0.105		0.0	0.978	
	Time^*^group	2.1	0.057		1.5	0.101	

*Results of repeated measures ANOVA of the mathematical difference between baseline and recovery (in minutes), by group; post-hoc: Tukey test. See also Figure 4. Bold typeface marks significant p-values*.

In a more detailed analysis, the differences between baseline and experimental day values were compared separately for each vigilance state in both control and hyperammonemic rats. This analysis revealed that the control animals exhibited a normal NREM recovery phase, lasting approximately 9 h with a gradual decline in time spent in NREM sleep (Figure 4A), while in hyperammonemic animals the primary recovery phase was shorter (as demonstrated by the statistical analysis described in Table 1), lasting only about 5 h (Figure 4B). In the first part of the recovery phase (h 9–16), the hyperammonemic animals exhibited significantly more wakefulness and less NREM sleep compared to the control animals (Table 1).

#### NREM bout duration during recovery sleep

Both control and hyperammonemic animals showed a significant increase in the duration of NREM bouts after sleep deprivation. The increase, however, was slightly lower in the hyperammonemia group compared to the controls [20 ± 3 s (+32%) vs. 26 ± 3 s (+43%)]. Higher time-resolution analysis showed a more pronounced increase in NREM bout duration in the first 4 h in both groups [hyperammonemia: 86 ± 15 s (158%); control: 88 ± 8 s (150%)]. In the control group, the increase was still present, albeit less pronounced, in the subsequent time-bin (4–8 h), while in the hyperammonemic rats the values dropped sharply to baseline.

### Adenosine

**Figure 6:**
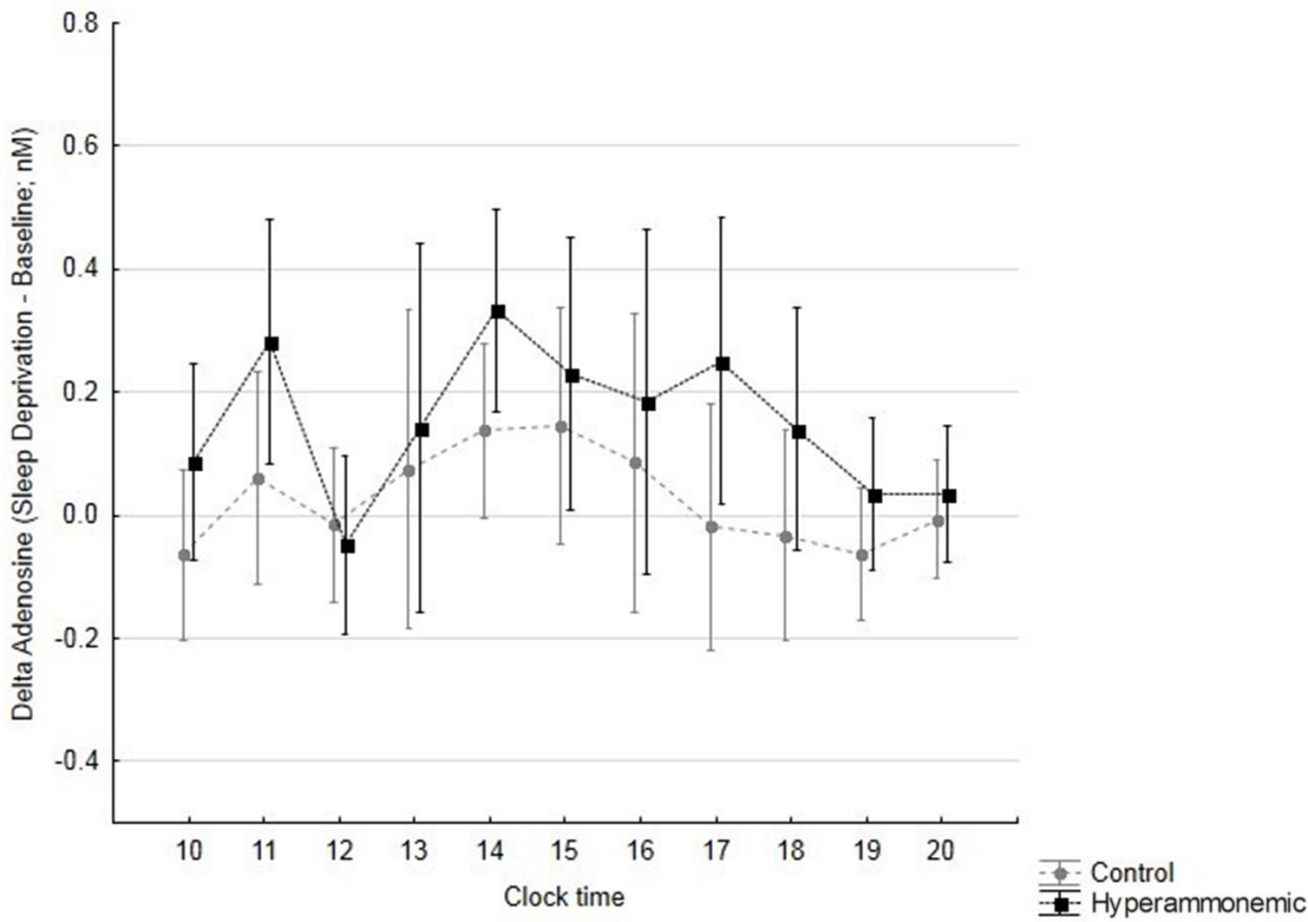
Differences in adenosine levels (sleep deprivation – baseline, with sleep deprivation starting at 11 h; mean ± 95% confidence interval) over time, in control (gray circles) and hyperammonemic (black squares) animals. *time: F* = 2.4, *p* = 0.012; *group: F* = 4.9, *p* = 0.047; time*group: F = 0.5, *p* = 0.829.

Out of 300 samples, 28 were lost for adenosine measurement. Average adenosine levels were lower (although not significantly lower) in hyperammonaemic compared to control animals at baseline (0.500 ± 0.064 nM vs. 0.683 ± 0.103 nM). Average adenosine levels rose during sleep deprivation, reaching similar values in hyperammonaemic and control animals (0.688 ± 0.103 nM vs. 0.740 ± 0.095 nM). Thus, the adenosine response to sleep deprivation was significantly greater and longer in hyperammonemic compared to control animals (Figure 6; *time: F* = 2.4, *p* = 0.012; *group: F* = 4.9, *p* = 0.047; time^*^group: F = 0.5, *p* = 0.829).

### Metabolites

**Figure 7:**
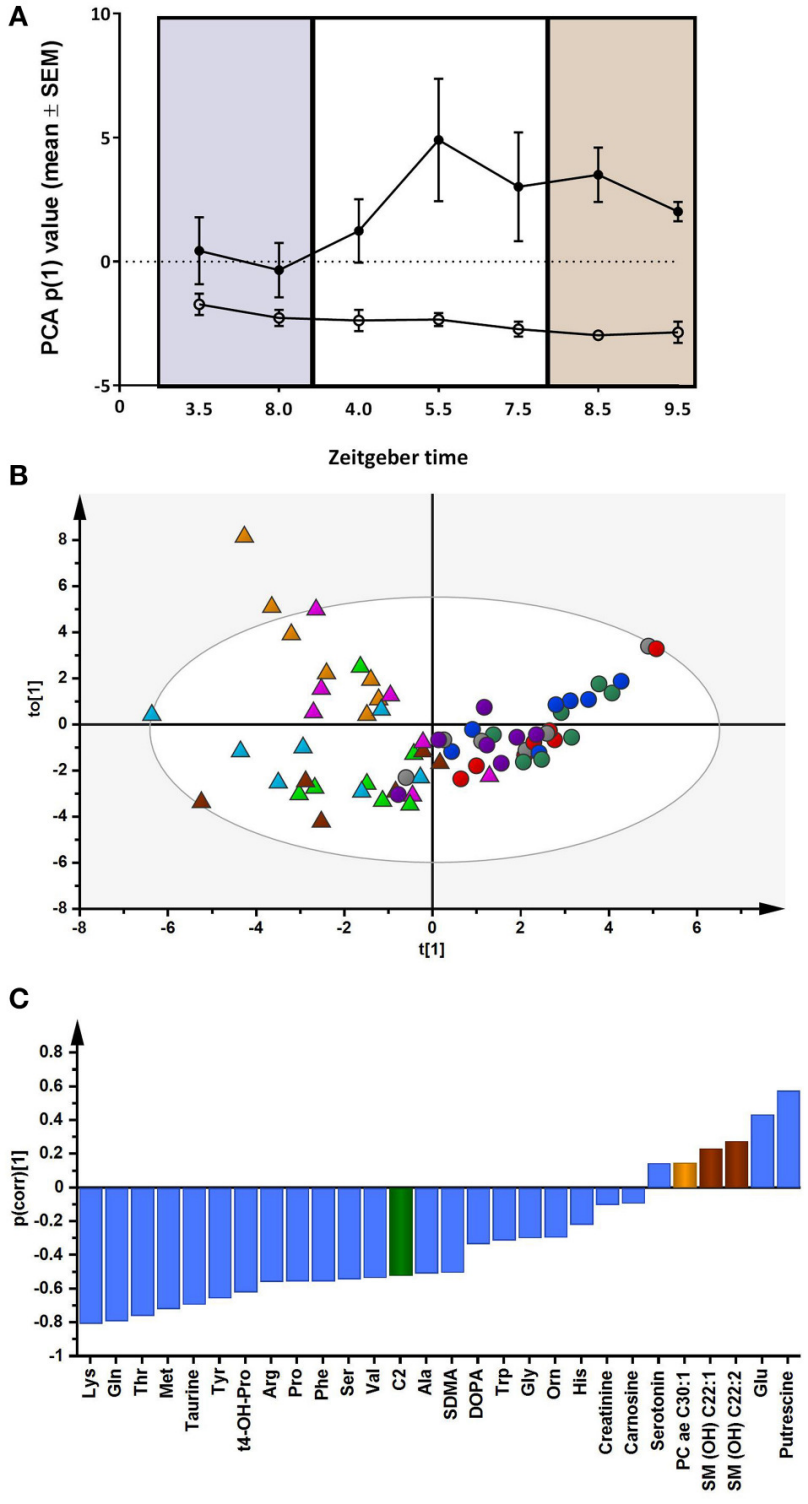
**(A)** Principal component analysis (PCA) of all the metabolite data (*n* = 28 metabolites). The change in mean score (±SEM) on PC1 in the control (*n* = 5, closed circles) and hyperammonemic animals (*n* = 5, open circles) across the time course (baseline, purple; during sleep deprivation, white; after sleep deprivation, orange) is shown. **(B)** Orthogonal partial least squares discriminant analysis (OPLS-DA) model showing separation by condition [Q2 (cumulative) = 0.575, R^2^X (cumulative) = 0.493; R^2^Y (cumulative) = 0.651]. Each animal is color coded; triangles represent control animals; circles represent hyperammonemic animals. **(C)** OPLS-DA *p*(corr) loading plot of control vs. hyperammonemic animals. Negative *p*(corr) values represent decreased and positive *p*(corr) values represent increased metabolite concentrations in hyperammonemic animals compared to controls. The metabolite bars are color coded according to metabolite class as follows: amino acids and biogenic amines (blue); acylcarnitines (green); phosphatidylcholine acyl-akyl (PC ae) (light orange); sphingolipids (SM) (brown).

Some metabolites were detectable (28 of 183, 15%) in the brain dialysate using targeted LC/MS metabolomics, largely comprising amino acids (*n* = 16) and biogenic amines (*n* = 8). PCA of the 28 metabolites showed a clear distinction between hyperammonemic (*n* = 5) and control animals (*n* = 5) in PC1 [amount of variance in the × matrix explained by PC1 (R^2^X) (cumulative) = 0.440, estimate of the predictive ability of the model (Q2) (cumulative) = 0.376]. A plot showing the mean score on PC1 (± SEM) for each sampling time point (*n* = 7) for the controls and hyperammonemic animals separately (*n* = 5 for both groups) is presented in Figure 7A. A clear difference between the controls and hyperammonemic animals across the time course (baseline; during sleep deprivation; after sleep deprivation) was observed. OPLS-DA models, validated by permutation analysis, showed separation between the control and hyperammonemic rats (Figure 7B). The p(corr) loading plot (Figure 7C) showed reduced concentrations of all of the amino acids (*n* = 16) except glutamate, as well as decreased taurine, t4-hydroxy-proline and acetylcarnitine levels in hyperammonemic animals. By contrast, putrescine and glutamate concentrations were higher in hyperammonemic animals. ANOVA analyses of the metabolite concentrations showed significantly reduced levels of 12 amino acids (alanine, arginine, glutamine, lysine, methionine, ornithine, phenylalanine, proline, serine, threonine, tyrosine, valine), taurine, t4-hydroxy-proline and acetylcarnitine in the hyperammonemic animals (FDR-adjusted *p* < 0.05). Only putrescine (FDR-adjusted *p* = 5.2E-10) and glutamate (FDR-adjusted *p* = 0.07) showed increased dialysate levels in the hyperammonemic animals compared to the controls. Of the 28 metabolites measured, only putrescine showed a significant group^*^time interaction. The p(corr) values for the OPLS-DA model are presented in Table 2.

**Table 2:** OPLS-DA loadings p(corr) values of the measured metabolites in models comparing controls with hyperammonemic animals.

**Metabolite**	***p*(corr)**
Lys	−0.805
Gln	−0.791
Thr	−0.758
Met	−0.719
Taurine	−0.693
Tyr	−0.655
t4-OH-Pro	−0.620
Arg	−0.558
Pro	−0.554
Phe	−0.554
Ser	−0.541
Val	−0.534
C2	−0.522
Ala	−0.507
SDMA	−0.504
DOPA	−0.334
Trp	−0.311
Gly	−0.297
Orn	−0.295
His	−0.219
Creatinine	−0.100
Carnosine	−0.092
Serotonin	0.148
PC ae C30:1	0.152
SM (OH) C22:1	0.235
SM (OH) C22:2	0.280
Glu	0.437
Putrescine	0.578

**Figure 8:**
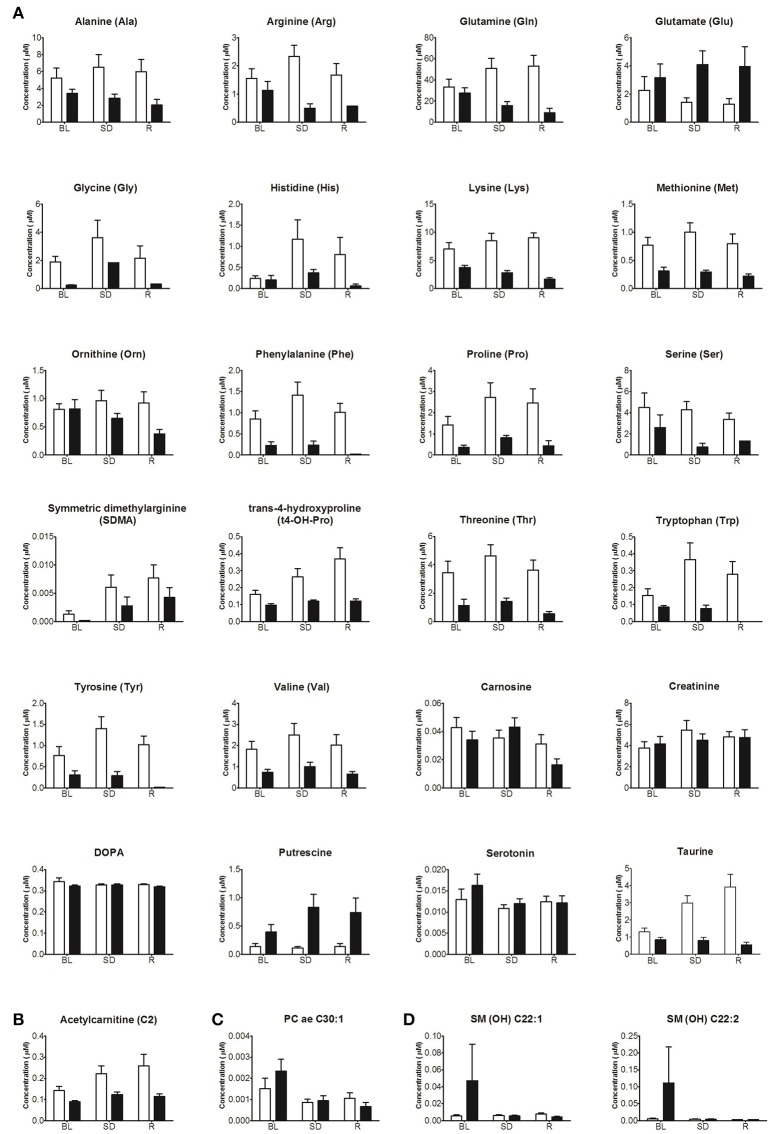
Concentrations (mean ± SEM) of the 28 metabolites at baseline (BL), sleep deprivation (SD) and recovery (R) in control (white) and hyperammonemic (black) animals. Metabolites are presented by class: **(A)** Amino acids and Biogenic Amines, **(B)** Acylcarnitines, **(C)** Phosphatidylcoline acyl-akyl, and **(D)** Sphingolipids.

For each animal used for the targeted metabolomics analysis (*n* = 5 in each group), seven time series samples were analyzed [2 at BL 3.5 and 8.0 ZT (Zeitgeber Time); 3 during SD 4.0, 5.5 and 7.5 ZT and 2 during recovery (R) following SD 8.5 and 9.5 ZT]. Figure 8 presents the mean (±SEM) concentrations of the 28 metabolites at BL, SD, and R in the control and hyperammonemic animals. Analyses comparing dialysate metabolite levels at BL, SD, and R revealed fewer significant differences, partly because of low sample number/samples below the limit of detection. As a result, the metabolites arginine, glutamate, glycine, histidine, phenylalanine, proline, serine, tryptophan, tyrosine, DOPA, and SDMA could not be analyzed.

Significant changes over time in relation to the different conditions (baseline/sleep deprivation/recovery) were observed for the metabolites acetylcarnitine, t4-hydroxyproline, PC ae 30:1 and carnosine (Figure 8). In addition, while glutamine and methionine increased during sleep deprivation in control animals, they did not in hyperammonemic animals (Figure 8).

## Discussion

The main findings of the present work were that prolonged hyperammonemia resulted in a tendency to decreased brain adenosine levels under a spontaneous sleep-wake cycle, whereas sleep deprivation increased basal forebrain adenosine levels more pronouncedly in hyperammonemic rats. In spite of the large increase in adenosine levels during sleep deprivation, the recovery sleep response was less efficient than normal, i.e., both shorter and more fragmented in the hyperammonemic animals.

The effects of increased ammonium levels on physiological functions are critically dependent on the dose and duration of the exposure (Felipo and Butterworth, 2002). Bjerring et al. (2015) have reported that rat brain slices exposed to ammonia exhibit a rapid increase in adenosine, with a linear dose-response correlation, while in the present study, basal forebrain adenosine levels were decreased under chronic hyperammonemia conditions. Increased ammonium levels have a multitude of effects on brain functions. As detoxification of ammonium takes place predominantly by astrocytes through glutamine synthesis, astrocyte swelling, changes in astrocyte morphology and expression of astrocyte proteins are frequent findings in hyperammonemia (Butterworth, 2010). Chronic hyperammonemia downregulates functional NMDA receptors and impairs NMDA-receptor-associated signal transduction pathways (Felipo and Butterworth, 2002), presumably decreasing NMDA-mediated neural activation. This tends to decrease energy consumption, which could at least partly explain the decreased adenosine levels during the spontaneous sleep-wake cycle in hyperammonemic animals.

Sleep deprivation increases adenosine levels in the basal forebrain of rats, which has been suggested to be one of the mechanism of sleep homeostasis (Porkka-Heiskanen et al., 1997). In the present study, the basal forebrain adenosine levels were increased during sleep deprivation in both control and hyperammonemic animals, the increase being larger in the hyperammonemic rats. However, while the control rats exhibited a normal, consolidated increase in slow wave sleep during recovery sleep, the recovery sleep response was shorter and more fragmented in the hyperammonemic rats, indicating that the adenosine response was not translated into a normal recovery sleep response.

Interestingly, chronic hyperammonemia decreases NMDA-mediated signal transduction through the NO-cGMP pathway, evidently by inhibiting the step by which NO increases cGMP (Felipo and Butterworth, 2002). During sleep deprivation, NO levels are increased (Kalinchuk et al., 2010). The large increase in adenosine levels in hyperammonemic animals during sleep deprivation, in spite of low levels during spontaneous sleep, could possibly be explained by the sleep deprivation-induced increase in NO levels in the basal forebrain.

Acute exposure of brain slides to ammonia rapidly increases adenosine (Bjerring et al., 2015). In the same study, *in vivo* recordings showed a tendency toward increased adenosine levels in rats with hyperammonemia and systemic inflammation compared to a control group (Bjerring et al., 2015). Our *in vivo* findings, over a longer period of time and defined sleep-wake conditions, suggest that chronic hyperammonemia results in enhanced, immediate adenosine release in response to sleep deprivation. This observation could explain a number of our previous observations in healthy volunteers with acute hyperammonemia, namely the increase in their subjective sleepiness (Bersagliere et al., 2012), slowing of their reaction times (Casula et al., 2015), and the deeper/longer subsequent sleep episode (Bersagliere et al., 2012). Effects in patients with cirrhosis were slightly different in response to the same stimulus (Bersagliere et al., 2012, 2013), most likely because induced hyperammonemia in cirrhosis is acute-on-chronic, with a mixture of immediate and adaptive effects.

Adenosine exerts its sleep promoting action mainly through binding to the A1 receptor (Halassa et al., 2009). Previous studies have demonstrated that mRNA levels of A1 adenosine receptor in the basal forebrain are up-regulated during sleep deprivation (Basheer et al., 2000) and are inhibited by an antisense oligonucleotide, or by a conditional knockout, resulting in an attenuated compensatory increase in NREM sleep time and slow wave activity (Thakkar et al., 2003; Bjorness et al., 2009). Functional imaging studies in cirrhotic patients have documented a decrease in cerebral levels of the A1 adenosine receptor (Boy et al., 2008).

Hyperammonemia did not affect baseline sleep time/architecture to any significant extent, at least over a 4-week period. This is in line with the results obtained by Llansola et al. (2012), who observed a decrease in REM sleep time only after 7 weeks of an ammonia-enriched diet. Analysis of the post-sleep deprivation recovery sleep highlighted a significant increase in NREM sleep time/decrease in wakefulness in both groups. However, in hyperammonemic animals the recovery sleep response was much shorter, in spite of the larger increase in adenosine levels. Similar impairment in generating restful sleep has been observed in cirrhotic patients after the acute induction of hyperammonemia (Bersagliere et al., 2012).

In an attempt to study the metabolic processes underlying central hyperammonemia and adenosine/sleep deprivation interactions, multiple metabolites were measured in the brain dialysate. All but one (glutamate) of the 16 amino acids had reduced dialysate levels in hyperammonemic animals. These abnormalities could reflect a number of processes, including impaired amino acid synthesis, increased catabolism or defective transport mechanisms, most likely involving different cell types (Benjamin and Quastel, 1975). An increase in extracellular glutamate content has been documented using different models of hyperammonemia/HE (Monfort et al., 2002), confirming that impaired glutamatergic neurotransmission contributes to the pathophysiology of the syndrome. It has been suggested that glutamate may act both as a nitrogen and energy buffer within the brain, thus its alterations may also be linked to those observed in the amino acids dialysate content.

Previously published literature mostly documents abnormalities in the levels of the branched chain amino acids (Soeters and Fischer, 1976; Tajiri and Shimizu, 2013), while we observed a reduction of virtually all amino acids measured. This may relate to the notion that chronic hyperammonemia results in brain atrophy, which may be mediated by disturbed protein synthesis/regeneration due to insufficient amino acid availability (Amodio et al., 2003; Garcia-Martinez et al., 2011). Our findings may have been confounded, to some extent, by the fact that animals fed the ammonia-enriched diet most likely ate less, and tended to grow less. This may also explain the discrepancy between the reduction of virtually all amino acids observed here and previous experimental (Okada et al., 2009) and clinical studies (Schmidt et al., 2004), especially in relation to glutamine, which has generally been observed to increase after exposure to ammonia. Insufficient amino acid availability may also affect the range of potential amino acid modulation in response to sleep deprivation/recovery, thus explaining the flat profiles observed in the hyperammonemic animals in this study. Our novel, preliminary findings also suggest that targeted metabolic profiling of brain dialysate offers promise to study brain amino acid metabolism in sleep-wake regulation.

A broader understanding of the underlying mechanisms could have been obtained if plasma metabolomics data were available, which is a limitation of our study. It should also be remembered that the model used in this study is a model of pure hyperammonemia (on healthy liver), thus some of the changes in protein metabolism due to hyperammonemia plus liver failure may not be present. On the other hand, the metabolic and sleep-wake effects observed solely by changing dietary ammonia are striking, and confirm that even in the absence of hepatic failure, hyperammonemia affects cerebral metabolism, neurotransmission and sleep-wake cycles. Hyperammonemic animals also had significantly reduced taurine, t4-hydroxy-proline and acetylcarnitine levels in their brain dialysate. A reduction in taurine was also recently observed in the cerebrospinal fluid of patients with hepatic failure and HE, while acetylcarnitine levels were increased (Weiss et al., 2016). Again, the discrepancy may be related to the presence of hepatic failure in the human study and its absence in our model.

Only putrescine was significantly increased in the brain dialysate of hyperammonemic animals. Increases in putrescine have been previously reported, for example, in the cerebrospinal fluid of a rat model of multiple sclerosis (Noga et al., 2012). In our model putrescine may be a correlate of either neuroinflammation (Rodrigo et al., 2010) or blood brain barrier alterations (Skowrońska and Albrecht, 2012). Of interest, putrescine increased further when hyperammonemic animals were sleep deprived. This finding suggests that the association of hyperammonemia and insomnia, which are both common in cirrhosis, may have more than additive effects.

In conclusion, hyperammonemia modulates the adenosine/metabolite/EEG response to sleep deprivation. These observations fit with both the excessive sleepiness and fragmented sleep exhibited by hyperammonemic patients.

## Author contributions

SMo, TP, DS, and RC conceived and planned the study; SMa, OS, and PS acquired the data; SMa, OS, SMo, BM, NC, TP, and DS analyzed the data; SMa, SMo, TP, DS, and RC drafted and reviewed the manuscript. The authors approved the final version and agree to be accountable for all aspects of the work.

### Conflict of interest statement

The authors declare that the research was conducted in the absence of any commercial or financial relationships that could be construed as a potential conflict of interest.
